# Development of bioactive SF/HAp/ZnO scaffolds incorporating succinic acid with potential bone regeneration capabilities

**DOI:** 10.1039/d5ra04926a

**Published:** 2026-02-17

**Authors:** Sadia Habib, Aysha Arshad, Hafsah Akhtar, Fahad Hussain Alhamoudi, Gülderen Karakuş, Gültekin Gökce, Aqif Anwar Chaudhry, Hamad Khalid, Ather Farooq Khan

**Affiliations:** a Interdisciplinary Research Centre in Biomedical Materials (IRCBM), COMSATS University Islamabad, Lahore Campus Defence Road, off Raiwind Road Lahore Pakistan ather.khan@aasu.edu.kw; b Dental Technology Department Applied Medical Science, King Khalid University Abha 62529 Kingdom of Saudi Arabia; c Department of Pharmaceutical Basic Sciences, Sivas Cumhuriyet University Faculty of Pharmacy 58140 Sivas Türkiye; d Department of Analytical Chemistry, Sivas Cumhuriyet University Faculty of Pharmacy 58140 Sivas Türkiye; e College of Engineering and Energy, Abdullah Al Salem University Kuwait

## Abstract

The challenges associated with using autografts for repairing bone defects have increased the demand for synthetic bone graft substitutes that can offer both structural support and biological activity. In this research, we developed a composite scaffold made of silk fibroin, hydroxyapatite, and ZnO (SF/HAp/ZnO), incorporating succinic acid (SA) as a functional additive to improve the physicochemical and biological properties of the scaffold. The morphology of the synthesized scaffold was confirmed by FESEM analysis, and a rougher morphology than pure SF was observed in the scaffolds. The study revealed that the scaffold displayed slow degradation (4.84% after 21 days in S4) and high swelling *in vitro*, and was hydrophilic in nature. A significant difference was observed in the specific strength of the scaffold. Importantly, SA acted as a modulator of surface chemistry, creating a favorable interface for cell attachment and proliferation. *In vitro* biocompatibility studies using calcein, DAPI, and PI staining (live/dead assay) confirmed that the SA-functionalized scaffold was non-toxic and supported high viability of MC3T3-E1 pre-osteoblasts after 48 hours. The findings indicated that the use of succinic acid delayed the degradation time. This multifunctional composite scaffold showed great potential for use in bone tissue engineering.

## Abstract 2

SF/HA/ZnO scaffolds with SA were fabricated *via* freeze gelation, exhibited good swelling, hydrophilicity, and controlled *in vitro* degradation and supported pre-osteoblast viability, indicating strong potential for bone regeneration.

## Graphical abstract

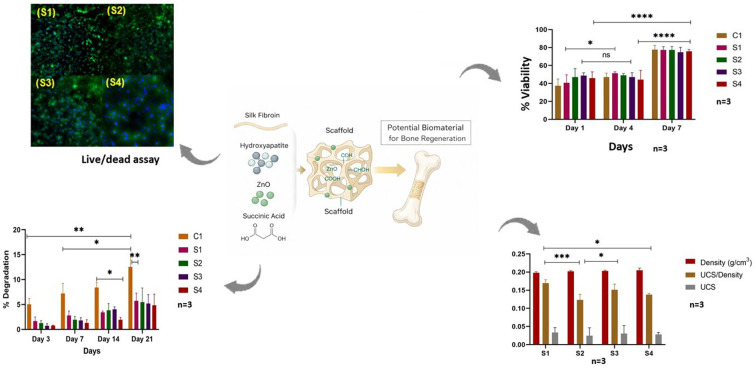

## 1 Introduction

Addressing bone defects presents a considerable challenge for surgeons, demanding innovative solutions for effective reconstruction. Disruption or deterioration of bones due to various factors, including tumour resection, trauma, congenital disorders, infections, or surgical interventions, can lead to significant bone loss, profoundly impacting both the physical and psychological well-being of individuals.^1,2^ Small bone defects generally heal naturally without the formation of scar tissue.^3^ However, circumstances such as critical sized-defects, infection, metabolic diseases, or aging can result in incomplete healing or non-union fractures.^4^ Currently, treatments like metallic implants, autografts, and allografts are considered the gold standard, but come with several drawbacks.^5,6^ For instance, the removal of metallic devices can cause secondary damage, autografts may lead to significant morbidity at the donor site due to limited bone availability, and allografts face challenges such as the risk of disease transmission and poor integration with host tissue. Consequently, there is an urgent need for more effective and stable treatments to address critical-sized bone defects.^7^

Tissue engineering (TE) has emerged as a promising avenue by replicating the intricate structures of living tissues,^8^ offering potential solutions in the domain of bone tissue engineering (BTE).^9^ The use of both injectable and non-injectable scaffolds has been widely researched in the field of bone-tissue engineering. Non-injectable scaffolds are created in a specific shape and composition to offer 3-D support to cells using various fabrication techniques.^10^

Amidst the diverse materials investigated for bone tissue engineering (BTE), natural polymers have gained attention owing to their biocompatibility, supporting cell attachment and proliferation akin to native tissues. Silk fibroin (SF) derived from *Bombyx mori* (silkworms) has exhibited remarkable biocompatibility *in vitro* and *in vivo*, coupled with adjustable biodegradability, superior mechanical strength, and minimal immunogenicity. These unique properties position SF as an excellent candidate for BTE applications.^11,12^ Furthermore, the fibrous architecture of SF closely mimics collagen type I (Col I) in bones, fostering bone regeneration by enhancing osteoconductive behaviour.^13^ SF also promotes the nucleation of nanocrystals of hydroxyapatite (HAp), resembling nano collagenous proteins (NCPs), thereby imparting bone-like characteristics.^12,14,15^

However, despite their biocompatibility, natural polymers often lack mechanical strength and inherent biological activity. To overcome this limitation, composite scaffolds comprising polymers and ceramics, notably calcium phosphates (CaPs) and synthetic HAp, have been developed to enhance mechanical properties.^16^ Synthetic HAp, resembling naturally occurring HAp in human bone, exhibits exceptional biocompatibility, osteoconductivity, chemical stability, and minimal immunogenicity.^9,11^ The tuneable microstructure of synthetic HAp aids in pore formation, facilitating bone tissue and blood vessel ingrowth.^9,17,18^

ZnO is well-known for its antibacterial, osteogenic, and angiogenic properties. ZnO accelerates a variety of biological processes and ultimately leads to the formation of new tissues.^19^ The *in vitro* biocompatibility^20,21^ and the influence of ZnO in promoting osteoblast growth has been well established.^22,23^

Succinic acid (SA), an important small biomolecule and an intermediate in the tricarboxylic acid (TCA) cycle,^24^ has been identified in compact bone, with approximately 3.6 mg of SA found per gram of bone.^25^ This suggests that it may play a significant role in the process of bone biomineralization. Research has shown that SA is effective in regulating calcium mineral formation and serves as a bioactive molecule for applications in hard tissue repair. It can chemically adhere to crystal surfaces by coordinating with calcium ions, influencing both the growth and structure of calcium minerals.^26,27^ Additionally, as a bioactive biomolecule, SA is often incorporated into bone substitute materials or medications, which can help decrease inflammation and promote the regeneration of bone tissue.^28^ Succinic acid-modified chitosan hydrogels have been used for neuronal differentiation and spinal cord injury repair in a study by.^29^ Succinic acid in this study increased the expression level of neuronal differentiation-related markers *in vitro* by 1.5-fold and enhanced spinal cord injury repair *in vivo*. Similarly, succinic and ferulic acid grafted chitosan hydrogels were used in another study for the repair of brain injury by.^30^

In this study, our focus lies on the fabrication and comprehensive characterization of SF, HAp, ZnO, and succinic acid (SA) composite scaffolds as a promising biomaterial supporting bone growth. Literature has reported the use of succinic acid for neuronal tissue engineering, but its role in bone tissue regeneration in combination with silk fibroin has not been reported. For the first time, SA is used in bone substitutes to support bone regeneration. SA, a naturally occurring compound found in animal and plant tissues, stands out in BTE due to its influence on cell growth and its non-toxic nature.^31–34^ The interaction between SF and SA potentially relies on hydrogen bonding between SA's hydroxyl (−OH) group and SF's amino (−NH_2_) groups. Our investigation aims to comprehensively evaluate the structural, chemical, mechanical, and biological properties of the resulting composite scaffolds. Furthermore, we aim to demonstrate the non-cytotoxicity and biocompatibility of these composite scaffolds, proposing their potential utility in bone repair and regeneration applications.

## 2 Materials and methods

Domestic silk moths, scientifically known as *Bombyx mori* cocoons were harvested by local farmers from Changa Manga Jungle, Punjab, Pakistan. HAp was kindly donated by the bone repair and regeneration group, IRCBM, Lahore, Pakistan. Cell culture supplies such as α-MEM supplemented with deoxyribonucleosides, ribonucleosides, and without ascorbic acid were purchased from Gibco (Catalog No. A1049001). Fetal bovine serum, trypsin, and phosphate-buffered saline were supplied by Thermofisher Scientific. Sodium hydroxide (NaOH) was purchased from Acros Organics, Propidium iodide and Phosphate-buffered saline tablets were purchased from Sigma-Aldrich, Germany. Calcein AM was ordered from Santa Cruz Biotechnology, and paraformaldehyde was supplied by Daejung. All the chemicals were used as received.

### 2.1. Fabrication of porous SF/HAp/ZnO/SA composite scaffolds

*B. mori* cocoons were processed to remove the immunogenic sericin portion. The previously reported method was used for the extraction and purification of SF.^35–37^ SF was used in a concentration of 4% (w/v) throughout the experiments for the fabrication of scaffolds. Table 1 shows the different compositions of porous scaffolds, each having different weight % of HAp, ZnO, and SA with respect to the SF. The solutions were homogenized and stored at −40 °C for 12 hours, followed by chilled ethanol treatment to remove water in the frozen state, and samples were placed at −40 °C for another 12 hours. The chilled ethanol was replaced with fresh ethanol every 24 hours. The complete process was repeated at least 3 times. At the final step, the scaffolds were repeatedly washed with deionized water to eliminate ethanol residues. The final scaffolds were cut into 10 mm diameter discs, followed by drying in an oven for 48 hours and vacuum drying for another 24 hours. The percentage of HAp, ZnO, and SA was calculated (w/w) with respect to SF (4%).

**Table 1:** Composition of porous SF/HA/ZnO/SA composite scaffolds. Hap, ZnO, and SA were added (w/w) with respect to SF (4%)

Sr.no	HAp%	ZnO%	SA%
C1	—	—	—
S1	40	—	—
S2	40	4	5
S3	40	4	10
S4	40	4	15

### 2.2 Physical characterization of fabricated composite scaffolds

#### 2.2.1 FTIR spectroscopy

The Nicolet 6700™ Thermo Fisher Scientific spectrometer, coupled with an ATR accessory, was used for the analysis. The Omnic™ software was used for FTIR data acquisition. The spectra were acquired in the 4000–650 cm^−1^ range and at a resolution of 8 cm^−1^. A total of 256 scans were obtained for each sample.

#### 2.2.2 Field emission scanning electron microscopy

The surface of freeze-gelated scaffolds was observed under a field emission scanning electron microscope. The scaffolds were cut into slices and sputter-coated with gold. Surface morphologies of the samples were performed using a variable pressure Field Emission Scanning Electron Microscope (FE-SEM) Apreo S from ThermoFischer Scientific, Eindhoven, The Netherlands.

#### 2.2.3 XRD

PANalytical Xpert powder diffractometer (Malvern, UK) was used to determine the phase composition and crystallinity of synthesized HAp and ZnO. The spectrum was acquired in the 2-theta range 20°–80°.

#### 2.2.4 Confocal laser scanning microscopic (CLSM) examination

To observe the microporous structure of the synthesized scaffolds, a confocal laser scanning microscope was used. Scaffolds were dyed with Calcein and DAPI, and subsequently imaged at 10×. 3D images of the scaffolds were obtained using a Z-stack. The region of interest was selected from z-plane images to include the microporous structure, beginning with the bottom section at least 1 mm above the surface of the scaffolds. Depth projection micrographs were obtained from horizontal sections from which 3D images were reconstructed.

#### 2.2.5 Porosity, density measurements, and mechanical testing

Mechanical properties were evaluated in relation to density and porosity. The liquid displacement method was used to measure the porosity and density of all the scaffolds. Samples of equal weight were cut, and the weights were noted as *W*. For density calculation, a known volume (*V*_1_) of ethanol was added to a measuring cylinder and covered. Pre-weighed samples were submerged in ethanol cylinders and placed in a vacuum for one hour. Vacuum forces out the air bubbles trapped in the pores, and these spaces are replaced by the ethanol. After one hour, the volume of the ethanol-submerged scaffold was noted (*V*_2_). The sample was removed from ethanol, and again the volume was noted (*V*_3_). Density was measured by using the following formula:
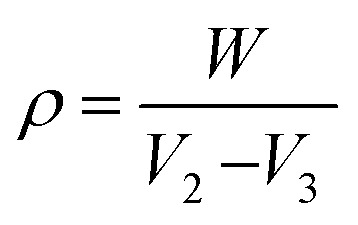


For the calculation of porosity, the following formula was used:
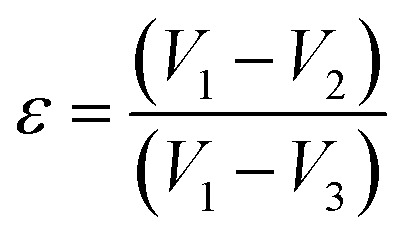


A dynamic mechanical analyzer (DMA 850, TA Instruments) was used in the compressive mode to determine the mechanical strength of the scaffolds. The measurements were carried out at 37 °C. The experiments were performed under a constant strain amplitude (50 µm). A small preload was applied to each sample to ensure that the entire scaffold surface was in contact with the compression plates before testing, and the distance between plates was equal for all scaffolds being tested. The samples were prepared in cylindrical form, having a height of 5 mm and a diameter of 3 mm. A stress–strain graph was used to evaluate the compressive strength.

#### 2.2.6 Contact angle

To determine the hydrophilicity or hydrophobicity of the scaffolds, contact angle analysis was done. A OneAttension version 4.1.3 (r9624) was used for contact angle measurements. Ultrapure water droplets were used, and analysis was performed at room temperature. A small amount of water was placed on scaffolds using micropipettes, and micrographs were captured.

#### 2.2.7 *In vitro* degradation studies

The samples were cut into equal dimensions and weight. Dry weight was calculated as *W*_o_, and samples were placed in well plates in triplicate. The samples were incubated at 37 °C in 2 ml PBS solution (pH 7.4). Before weighing, the samples were first rinsed with ultra-pure water, followed by vacuum drying (atmospheric pressure) at room temperature. The samples were weighed on days 3, 7, 14, and 21, respectively. The percentage degradation was calculated using the following formula, Degradation (%) = (*W*_o_ − *W*_*t*_ / *W*_o_) × 100*W*_0_ = initial weight of the sample, *W*_*t*_ = final weight of sample at respective time.

#### 2.2.8 Swelling studies

The study was planned to evaluate the swelling performance of fabricated scaffolds for 0, 3, 7, and 14 days in PBS solution at 37 °C.^38^ Equal-weighted discs were cut, and dry weights of all discs were recorded as *W*_d_, followed by immersion of the discs in PBS for 0, 3, 7, and 14 days. The scaffolds were removed at the respective times. The dripping water was removed with the help of filter paper and weighed as *W*_s_. The following equation was used for swelling ratio calculations.Swelling ratio = (*W*_s_ – *W*_d_)/*W*_d_Percentage water uptake = (*W*_s_ – *W*_d_)/*W*_d_ × 100

### 2.3 *In vitro* biological characterization of the fabricated composite scaffolds

#### 2.3.1 Cell culture

The ATCC® CRL-2594 subclone 14 MC3T3-E1 preosteoblast cells were used for cell culture evaluations. Culture conditions comprised of maintaining cells in a 5% CO_2_ humidified incubator at 37 °C. The alpha-MEM (supplemented with nucleosides & no ascorbic acid), substituted with 1% penicillin/streptomycin and 10% FBS, was used. Upon reaching 90–100% confluency, cells were sub-cultured by using trypsin under sterile conditions according to standard protocol. Complete media was replenished every second day. Cells of passage 9 were used for all the experiments, and all the experiments were run in triplicate. The samples were sterilized using gamma radiation.

#### 2.3.2 Cell viability assay

Alamar blue assay (Alamar BioSciences, Sacramento, CA), a colorimetric assay, was performed to evaluate the viability of pre-osteoblast cells on the SF/HAp/ZnO/SA composite scaffolds. 5000 cells were calculated and seeded onto the surface of the scaffolds. The resulting mixture was incubated in a humidified incubator at 37 °C with 5% CO_2_. Alamar blue readings were measured on days 1, 4, and 7 for all the samples. For each reading, 100 µL Alamar blue reagent was added to the well, followed by incubation for two hours and thirty minutes. The absorbance was measured using a Microplate reader (Labtech LT4500) at 570 nm.

#### 2.3.3 Live-dead assay

Calcein AM, DAPI, and propidium iodide were used for this assay. 5 × 10^4^ cells were seeded on the surface of each scaffold under sterile conditions. The samples were incubated in 5% CO_2_ humidified incubator at 37 °C for 30 minutes to facilitate the adhesion of cells. This was followed by adding complete media. The scaffolds were further incubated for 48 hours. Calcein AM, DAPI, and propidium iodide (4 µM) were added to each scaffold, and a confocal laser scanning microscope was used for imaging. All the images were taken at 10×.

### 2.4 Statistics

All the experimental results were expressed as the mean ± standard deviation. A series of two-way ANOVA was performed using GraphPad Prism (GraphPad Software, Version 9.5.1) to evaluate the significant difference. Tukey's multiple comparison test was performed to evaluate the statistical differences in the fabricated scaffolds. All experiments were performed in triplicate.

## 3 Results and discussion

### 3.1 FTIR

**Fig. 1:**
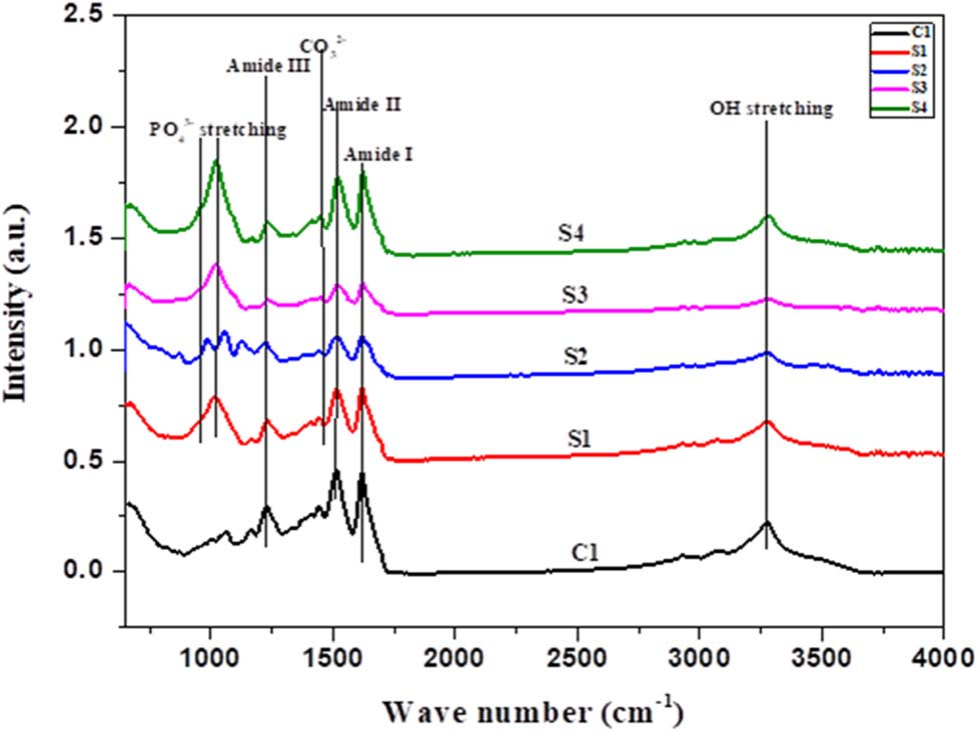
FTIR spectra of composite scaffolds C1, S1, S2, S3, S4, showing the characteristic functional groups.

The interactions between SF, HAp, ZnO and SA after composite fabrication were evaluated by FTIR (see Fig. 1 & Table 2). At 1621 cm^−1^ and 1222 cm^−1^ bands of amide-i (C=O stretching) and amide-iii (C–N stretching and C=O bending) were observed, respectively, due to random coil conformation.^39^ Whereas β-sheet formation was confirmed due to the presence of amide ii (C–H and N–H) group peak at 1512 cm^−1^.^40^ These results confirmed that β-sheet and random coil structures of SF were coexisting in the fabricated samples. Moreover, successful incorporation of HAp into SF was confirmed by the appearance of the characteristic bands of HAp for different modes of vibration for PO_4_^3−^. A clear characteristic band at 1056 cm^−1^ was attributed to asymmetric stretching of P–O in PO_4_^3−^ of HAp.^41^ A shoulder band at 970 cm^−1^ represented the symmetric stretching mode of P–O.^42,43^ The OH- stretching vibration band is also present at 3270–3278 cm^−1^.^13,44^ Presence of a small band at 1444 cm^−1^ indicated the presence of CO_3_^2−^ groups which suggested the presence of B-type carbonate apatite along with pure apatite, originated from the absorption of CO_2_ from the atmosphere.^13,45^ B-type carbonated apatite is found in young bones, which could help bone maturation.^13,46^ It can also be observed that the peaks of each fabricated composite scaffold are broadened due to the addition of SA as compared to the control (C). This broadening and sharpening of OH and amide i, ii, and iii peaks can be due to the increased H-bonding due to the association of –OH from either HAp or SA and between –NH groups of SF molecules. The steric hindrances of the polymeric association network might also have caused peak broadening.^24,26^ The FTIR spectra have provided evidence of the successful incorporation of SA. The spectra show the characteristic peak of SA, which confirms there are no significant changes in the chemical structure of the composite.

**Table 2:** FTIR vibrational bands of composite scaffolds, along with their respective wavenumbers

Vibrational bands	Wavenumbers (cm^−1^)
Symmetric P–O stretching	970
Asymmetric P–O stretching	1056
Amide-i	1621
Amide-ii	1512
Amide-iii	1222
CO_3_^2−^ stretching	1444
O–H stretching	3270–3278

### 3.2 FESEM

**Fig. 2:**
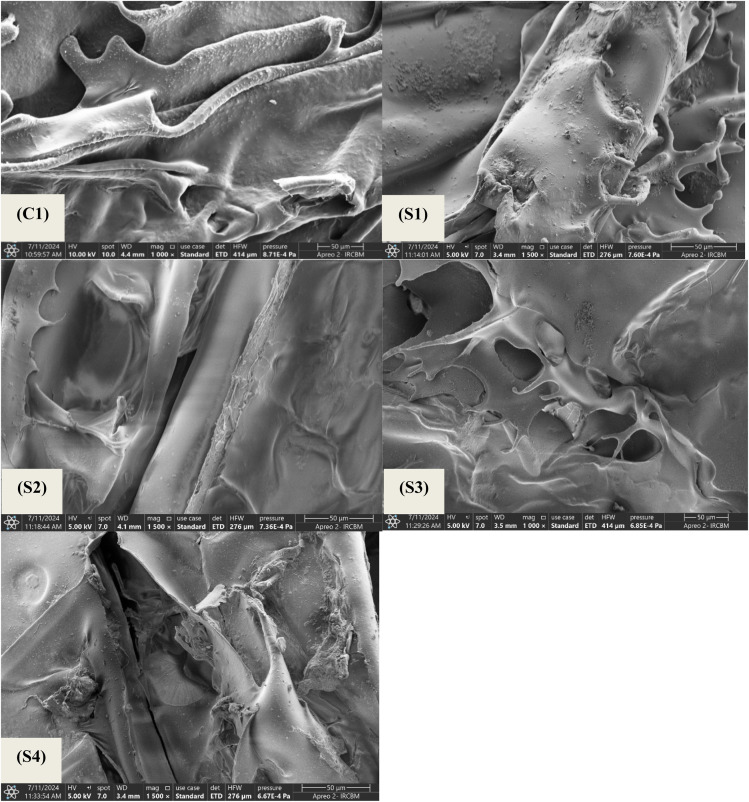
C1 : SF, S1 : SF/HAp, S2 : SF/HAp/ZnO/SA 5%, S3 : SF/HAp/ZnO/SA 10%, S4 : SF/HAp/ZnO/SA 15%.

FESEM analysis is an accurate method for studying the surface features of scaffolds. The morphology of scaffolds was examined by field emission scanning electron microscopy. Fig. 2(C1–S4) shows the morphology of scaffolds (control and samples). Fig. 2(C1) represents the morphology of pure SF scaffold, whereas (Fig. 2(S2–S4)) represents the morphology of SF/HAp/ZnO/SA scaffolds with varying concentrations of SA. The FESEM images reveal the smooth structure of the pure SF membrane. Moreover, the substituent scaffolds of SF modified with HAp, ZnO, and SA, also have the same morphology. The addition of these constituents did not alter the architecture of membranes; however, a roughness in surface morphology was observed by the addition of HAp as shown in Fig. 2. The presence of HAp on the surface is beneficial for cell proliferation and adhesion, since HAp is well known for its biocompatibility. Similar results have been reported in the literature which show that the addition of polymeric materials in the freeze-dried SF membranes did not change its morphology. As documented previously,^47^ the addition of TiO_2_ nanoparticles in SF freeze dried membranes did not alter the architecture of pure SF membranes.

### 3.3 XRD

**Fig. 3:**
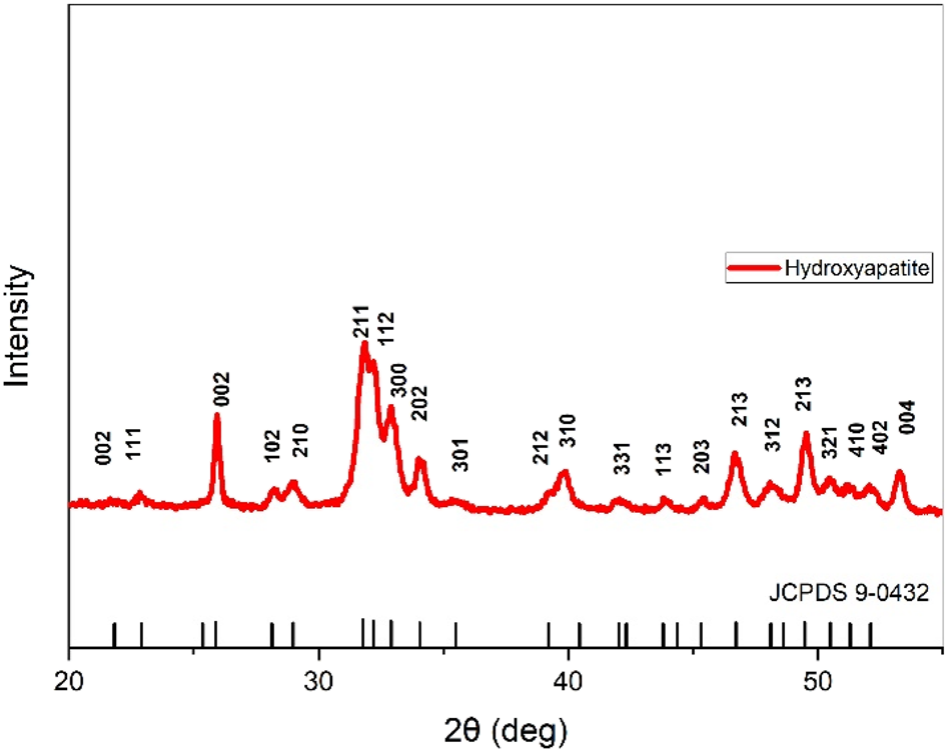
XRD spectrum of hydroxyapatite.

**Fig. 4:**
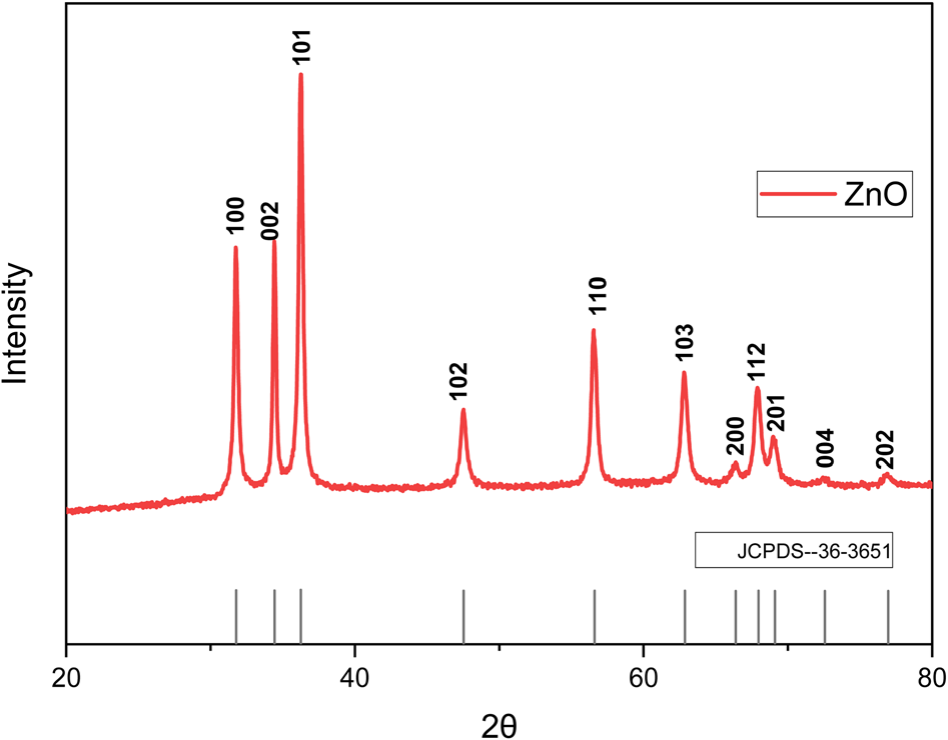
XRD spectrum of zinc oxide.

The XRD analysis for HAp and ZnO is shown in Fig. 3 and 4, respectively. The diffraction peaks in the XRD patterns were in good agreement with the pure hexagonal phase (JCPDS card # 09–0432).^48^ Specifically, the peaks at 2*θ* values of 22.88, 25.88, 31.74, 32.88, 34.06, 39.26, 43.82, 49.44, 51.22 correspond to the (111), (002), (210), (300), (202), (212), (113), (213), (410) and (004) planes, respectively. The XRD results suggest a predominant phase linked to the hexagonal symmetry associated with pure crystalline nanometer-sized HAp. The XRD of ZnO nanoparticles confirmed the successful synthesis of pure zinc oxide nanoparticles. The sharp diffraction peaks of the synthesized products indicates their good crystallinity. The diffraction peaks in the XRD pattern exhibited a hexagonal crystal structure of ZnO nanoparticles (JCPDS card # 36-3651). The diffraction peaks at the crystal planes (100), (002), (101), (102), (110), (103), (200), (112), (201) and (004) indicated that the samples were polycrystalline in nature.^49^

### 3.4 Confocal laser scanning microscopic (CLSM) examination

**Fig. 5:**
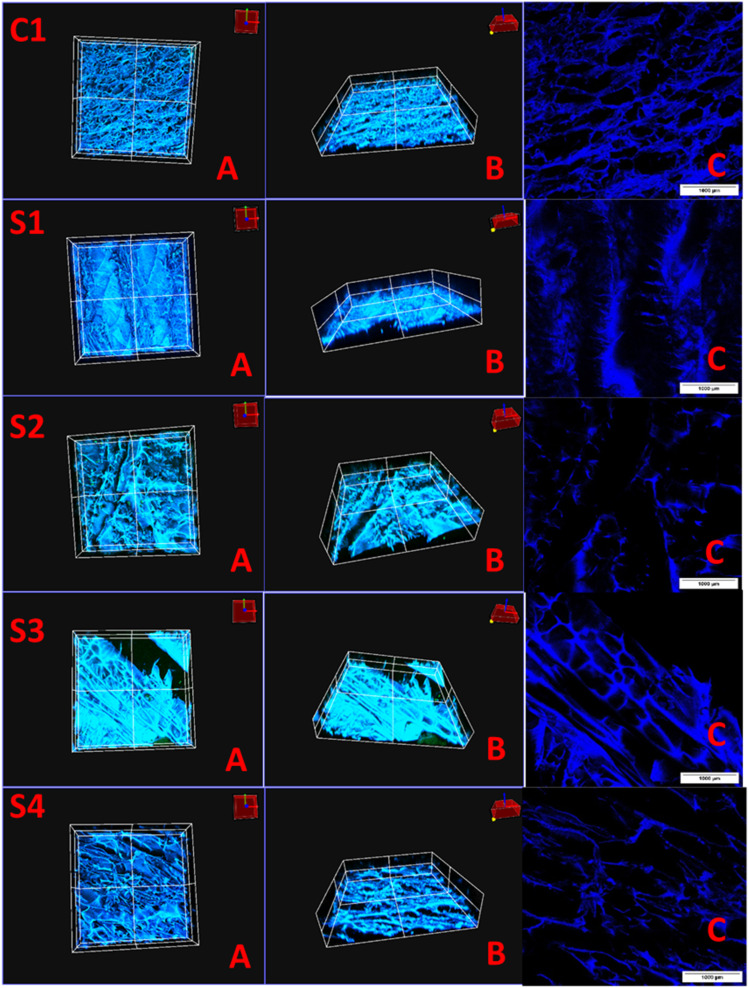
Confocal laser scanning microscopy (CLSM) images of freeze-gelated silk-based scaffolds. The left two columns show z-stack reconstructions highlighting the three-dimensional porous microarchitecture, while the right column presents DAPI-stained images revealing the fibrillar silk framework. Images were obtained at 10×. Scale bar shows 1000 µm.

Confocal laser scanning microscopy (CLSM) was utilized to examine the internal microstructure of freeze-gelated silk-based scaffolds. The first two columns (A and B) of Fig. 5 display reconstructed z-stack images, offering a three-dimensional view of the scaffold's porosity and connectivity, while the right column shows DAPI-stained images that enhance the visualization of the structural framework. Calcein staining was employed to highlight the mineral-binding regions of the scaffolds, with DAPI adding further structural contrast. The z-stack reconstructions clearly demonstrate that all scaffolds have an interconnected microporous structure, which is essential for promoting cellular infiltration, nutrient transfer, and eventual vascular growth. Notable variations between the control (C1) and modified scaffolds (S1–S4) are evident, as some formulations exhibit more open and connected pores, whereas others have denser areas with less pore interconnectivity. The non-destructive capability of CLSM to scan hydrated scaffolds allowed for a more accurate representation of pore morphology in comparison to traditional SEM imaging, which often necessitates dehydration and can compromise fragile pore structures. The DAPI-stained images (right column, C) further support the presence of a continuous porous framework within the silk fibroin matrix. The blue fluorescence outlines the fibrous nature of the scaffolds, indicating that the freeze-gelation method maintained the intrinsic silk microfibrillar structure. The CLSM findings confirm the highly porous and interconnected networks of freeze-gelated silk-based scaffolds, making them suitable for tissue engineering applications. These microstructural characteristics are crucial, as they directly affect cell migration, osteogenic differentiation, and new blood vessel formation in bone tissue engineering. The simultaneous use of calcein and DAPI facilitated the visualization of both mineral-affinitive regions and fibrous protein structures, resulting in a thorough evaluation of scaffold microarchitecture.

### 3.5 Contact angle measurement

**Fig. 6:**
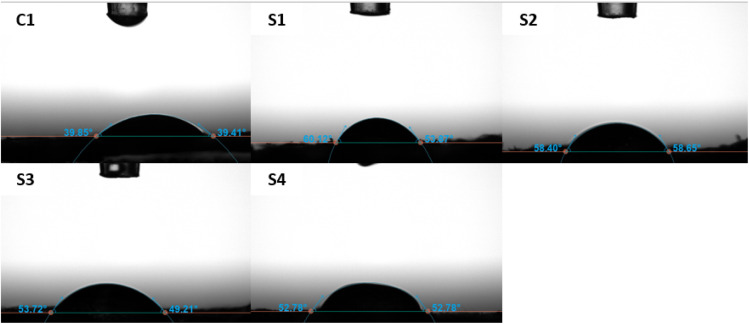
Contact angle images of scaffolds C1, S1, S2, S3, and S4.

**Fig. 7:**
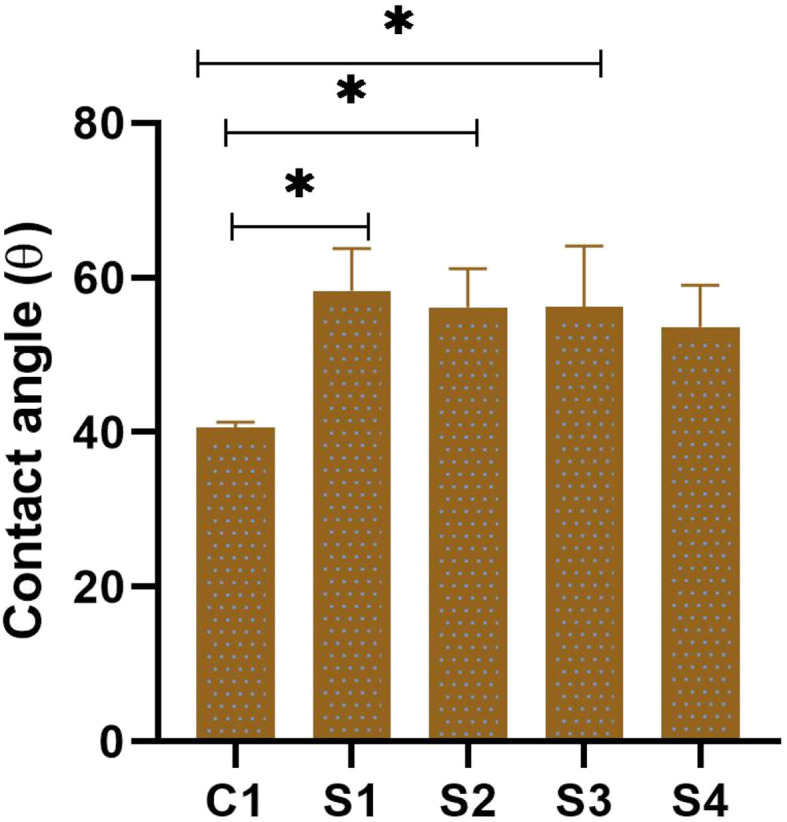
Contact angle measurements of scaffolds where error bars indicate standard error (*n* = 3) *p < 0.1.

The hydrophilicity and surface wettability of polymeric films, indicated by the water contact angle, can directly impact protein adsorption and cell attachment. Therefore, the interaction between cells and scaffolds relies heavily on the hydrophilicity of the polymeric matrix. We examined the surface properties and wettability of the samples by assessing the water contact angle. Fig. 6 and 7 represent the contact angle images and measurements of the prepared scaffolds. The measured contact angle for the C1 scaffold was 40.573° ± 0.750, which shows the hydrophilic nature of pure SF scaffolds. A smaller contact angle of pure SF indicates that the scaffold is more hydrophilic, which can promote cell proliferation and adhesion.^50^ When a water droplet strongly interacts with a hydrophilic SF, it quickly wets the surface due to the capillary effect, causing the surface to become hydrophilic.^51^ The addition of HAp in the silk scaffolds increased the contact angle of the scaffolds, and the measured contact angle for S1 was 58.313° ± 5.444. This result correlates with the fact that HAp is hydrophobic^52^ and explains the increase in contact angle of the scaffold. Similarly, with the addition of SA, an increase in scaffold contact angle was observed. The measured contact angles for S2, S3, and S4 were 56.147° ± 5.007, 56.263° ± 7.861, and 53.620° ± 5.418, respectively. The addition of SA in SF slightly enhanced the contact angle of the scaffolds. The increase in contact angle in S2, S3, and S4 scaffolds can be attributed to the presence of ZnO. Various studies have reported an increase in water contact angle upon the addition of ZnO nanoparticles in the scaffolds.^53^ The optimal contact angle for a cell adhesive material should be between 55° and 75°.^17^ The findings indicate that all scaffolds exhibit favorable wetting properties.

### 3.6 *In vitro* degradation studies

**Fig. 8:**
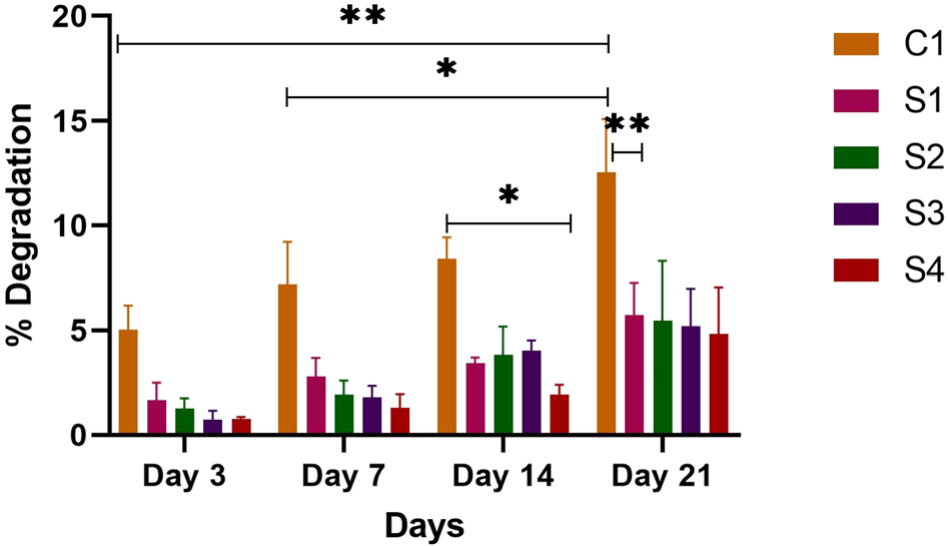
Mass loss% of SF/HAp/ZnO/SA composite scaffolds where error bars indicate standard error (*n* = 3). The significant mass loss is represented with *. *p < 0.1, **p < 0.01.

The primary requisite of biomaterials is that their biodegradation rate coincides with the new tissue regeneration process, to provide enough time to facilitate the new bone formation by allowing nutrient transport.^54–56^ A scaffold that breaks down slowly could prevent the formation of new tissue, whereas one that breaks down too rapidly might not provide enough mechanical support over time.^57^ Therefore, the degradation of all the scaffolds was measured in PBS over a period of 21 days. The concentration of polymers, SA, and the time duration of soaking samples in PBS solution affect the rate of degradation of the scaffolds. After the particular time period, all scaffolds exhibited some weight loss, which increased consistently over the 21 days period (see Fig. 8). This shows the controlled and gradual degradability of scaffolds. C1 showed the greatest degradation in all scaffolds whereas with the addition of HAp, ZnO and SA, the rate of degradation decreased as compared to C1. The degradation of C1 was due to hydrophilic interactions between SF and PBS.^58^ Among S2, S3 and S4 scaffolds having 5,10 and 15% SA, respectively, S4 had less degradation, followed by S3 and then S2. With the increasing concentration of SA, the rate of degradation decreased. It is noteworthy that with the incorporation of 4% HAp, the degradation rate decreased as compared to C1 at 3,7,14 and 21 days. The decreased rate of degradation of SF composite due to the addition of HAp was also reported by Rama & Vijayalakshami.^52^ The percentage degradation rate of the scaffolds C1, S1, S2, S3 and S4 after 21 days was 12.54%, 5.73%, 5.47%, 5.20 and 4.84% respectively. There was a significant degradation between S4 and C1 at days 7 and 14. Similarly, at day 21, scaffolds S1–S4 showed significant differences as compared to C1. Over time, the breakdown of scaffolds leads to the release of the constituent particles, allowing them to exert their influence. Moreover, the released HAp particles are osteoconductive, and they may interact with cells to encourage the migration of osteoblasts.^59,60^ The results reveal that the scaffolds show appropriate degradability for bone tissue regeneration applications.

### 3.7 Swelling studies

**Fig. 9:**
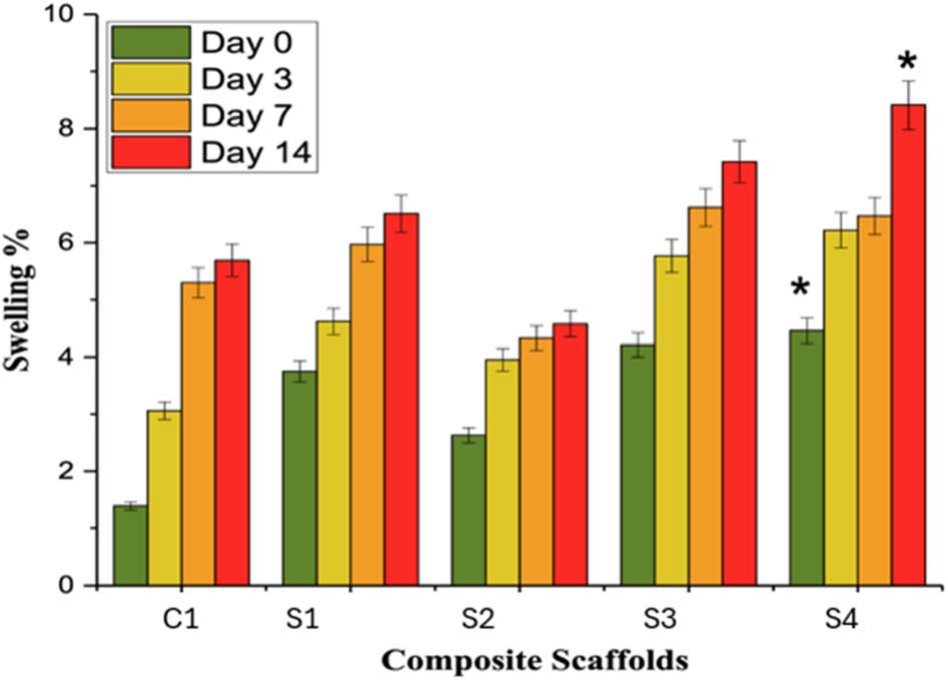
Swelling% of SF/HAp/SA scaffolds, where error bars indicate standard error (*n* = 3) *p < 0.1.

The swelling property of a biomaterial is very important for transporting minerals and nutrients to boost the bone growth process. Additionally, most natural polymeric bone repair scaffolds display swelling behavior when immersed in biological fluids. The swelling behavior of fabricated scaffolds was assessed in PBS at pH 7.4 for 0, 3, 7, and 14 days, respectively (see Fig. 9). The data showed a gradual increase in swelling behavior with the introduction of HAp in SF, and a further increase in swelling trend was observed with the addition of different concentrations of SA. A similar trend was observed for all the composite scaffolds till day 14. This pronounced increase in the swelling behavior is because of the highly hydrophilic surface due to the presence of SF. HAp and SF possess the –NH_2_ and –OH groups (hydrophilic groups), which are involved in assisting water absorption, hence resulting in increased swelling behavior of the composite scaffolds. The water-absorbing ability of the scaffolds was gradually increased from C1 to S4, possibly due to increased porosity, which resulted in increased capillary action.^61^ The existence of small pores in the composite scaffolds is responsible for the swelling, which promotes cell attachment.^13,62^ The swelling nature of composite scaffolds is dependent on the hydration of hydrophilic groups as well as hydrophobic groups.^63^

### 3.8 Mechanical properties

**Fig. 10:**
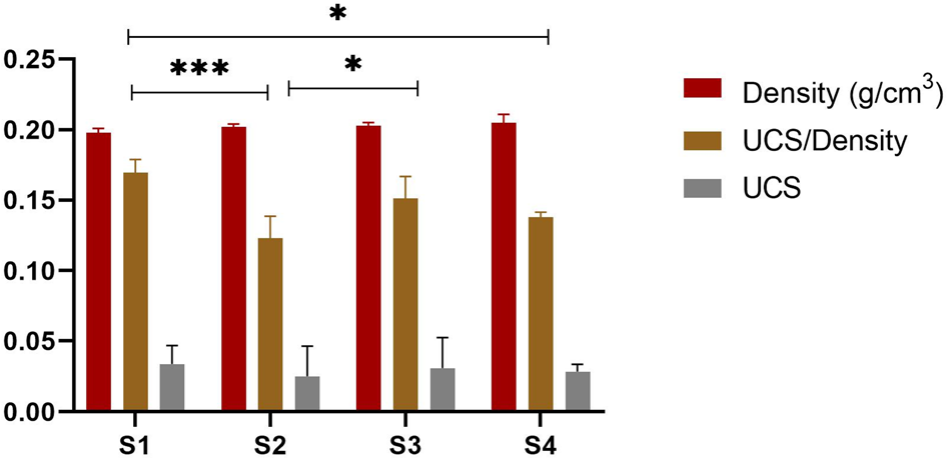
Density, UCS, and specific compressive strength of scaffolds. *p < 0.1, ***p < 0.001 (*n* = 3).

**Fig. 11:**
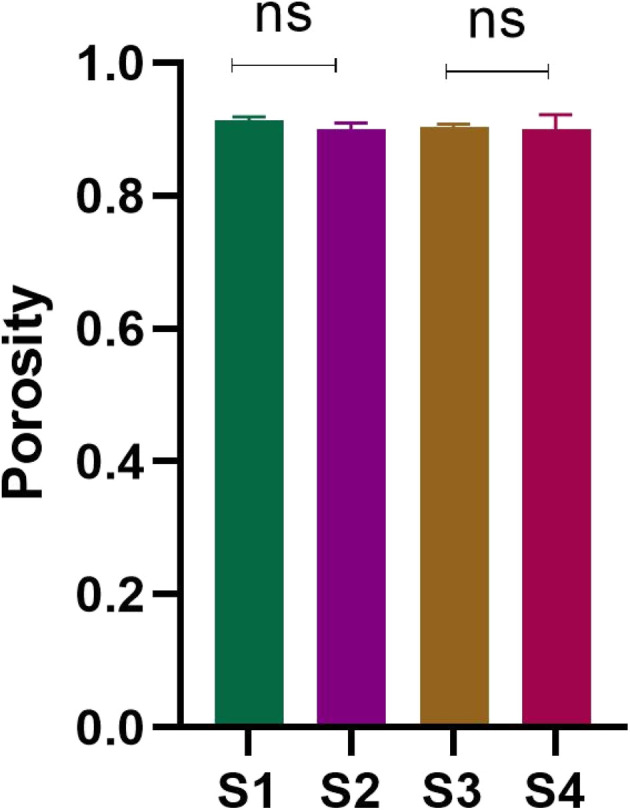
Porosity of composite scaffolds S1, S2, S3 and S4 (*n* = 3).

**Fig. 12:**
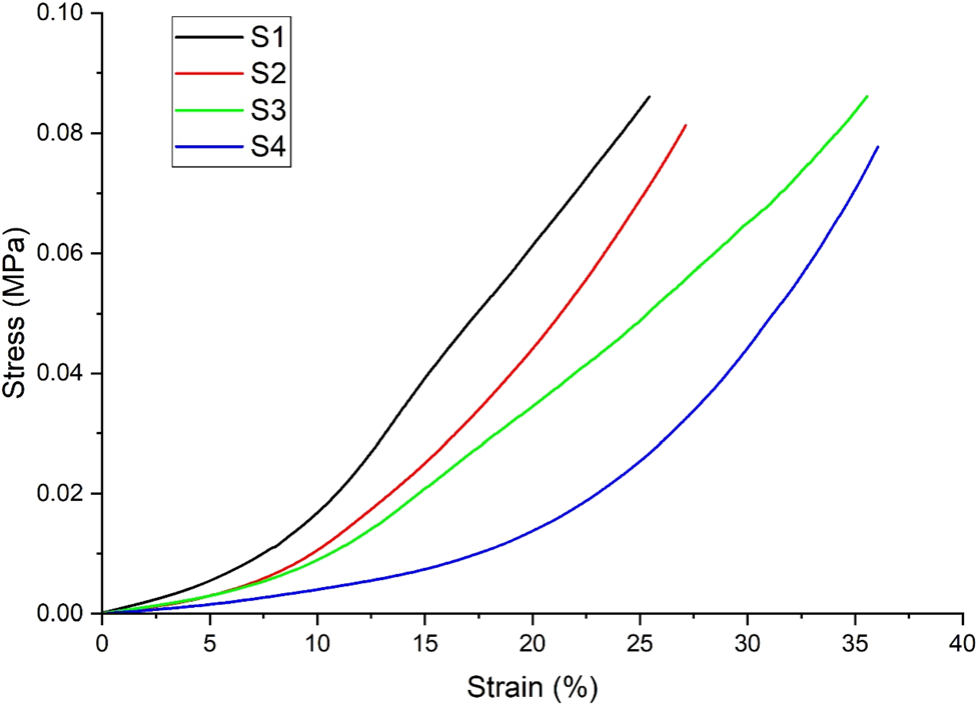
Graphical representation of stress–strain behavior of synthesized scaffolds (*n* = 3).

The mechanical strength of an ideal bone graft should be similar to that of natural bones. The mechanical strength of scaffolds is usually influenced by the size of the pores and porosity, but the contraction power of scaffolds is dependent on the composition of composites as well. DMA analysis in the compression mode was performed to evaluate the mechanical properties of the synthesized scaffolds. All the scaffolds were analyzed for mechanical strength through a stress–strain curve and in relation to density and porosity. The stress–strain curve provides information about the break point of composite materials. Fig. 12 shows the stress–strain curves for the scaffolds investigated. The values of Young's modulus were calculated from the stress–strain graph for each scaffold as listed in Table 3. The Young's modulus for the S1 (SF/HAp) scaffold was 0.00258 ± 0.0000473 MPa. Whereas S2, S3, and S4 scaffolds having different concentrations of SA showed the Young's modulus of 0.00189MPa ± 0.0000406, 0.00188MPa ± 0.0000343, and 0.0012 ± 0.0000297 MPa, respectively. From the data, it could be inferred that the compressive strength of the scaffold is related to the composition, porosity, and density of the scaffolds, listed in Table 3. Fig. 10 shows the graph of density, ultimate compressive strength (UCS), and specific strength of scaffolds, whereas the porosity is depicted in Fig. 11. The density of the sample S1 was 0.198 ± 0.003, whereas it was 0.202 ± 0.002, 0.203 ± 0.002, and 0.205 ± 0.006 for S2, S3, and S4, respectively. No significant difference was exhibited in the densities of the scaffolds. A similar trend was observed for the porosity of the scaffolds, which showed a non-significant difference. No significant difference was observed in the ultimate compressive strength (UCS) of the synthesized scaffolds. However, there was a significant difference in the specific strength of the scaffolds. S1 scaffold without the addition of any SA showed the highest specific strength of 0.169 ± 0.009254 MPa cm^−3^. A similar trend in the mechanical behavior of freeze-dried chitosan/hydroxyapatite cross-linked hydroxypropyl methyl cellulose scaffold has been previously reported.^64^ The ultimate compressive strength and Young's modulus of the scaffolds are significantly lower than those of natural bone. Cortical and trabecular bone exhibit compressive strengths of 100–230 MPa and 2–40 MPa, respectively.^65^ Several studies have reported compressive strengths for silk fibroin-based porous scaffolds fabricated by freeze drying or related processes. For example, silk fibroin/ostacalcium phosphate-based polydopamine-coated biodegradable scaffolds exhibited compressive strength of 65.66 ± 5.45 kPa, while pure SF scaffold showed the compressive strength of 38.18 ± 12.53 kPa.^66^ Due to the low mechanical strength of these scaffolds, they are intended to be used for applications involving non-load-bearing or low-load-bearing bone defects, prioritizing biological performance instead of high load-bearing strength (Fig. 12).

**Table 3:** Compressive strength, Young's modulus, density, and porosity of scaffolds

Sample code	Composition	Density (g cm^−3^)	Porosity%	Young's modulus (MPa)	UCS (MPa)	UCS/Density MPa (g^−1^ cm^−3^)
S1	SF/HAp/ZnO	0.198 ± 0.003	91.414 ± 0.004	0.00258 ± 0.0000473	0.03364 ± 0.01309	0.169642 ±
						0.009254
S2	SF/HAp/ZnO/5% SA	0.202 ± 0.002	89.932 ± 0.01012	0.00189 ± 0.0000406	0.02485 ± 0.02141	0.123203 ±
						0.01514
S3	SF/HAp/ZnO/10% SA	0.203 ± 0.002	90.303 ± 0.005	0.00188 ± 0.0000343	0.03079 ± 0.02167	0.151451 ±
						0.01532
S4	SF/HAp/ZnO/15% SA	0.205 ± 0.006	90.025 ± 0.022	0.0012 ± 0.0000297	0.02822 ± 0.005148	0.137659 ±
						0.003640

### 3.9 Cell viability assay

**Fig. 13:**
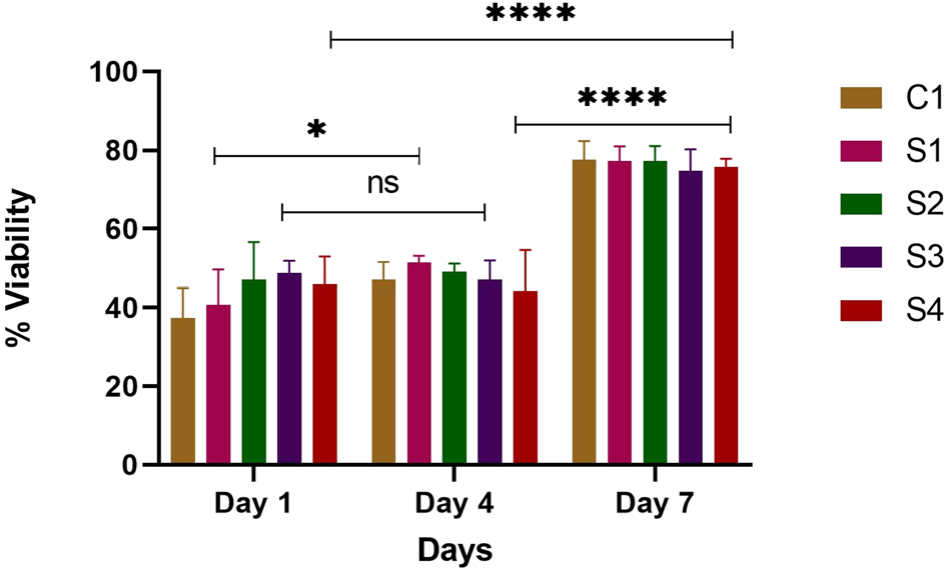
% Age pre-osteoblast cell viability of scaffolds, where error bars indicate standard error (*n* = 3). Error bars show standard error. *p < 0.1, ****p < 0.0001.

The metabolic activity of a cell is the primary marker to estimate its viability. Quantitative cell viability assay, using pre-osteoblast cells, was determined by performing the Alamar blue assay. Fig. 13 shows a bar graph of cell viability on scaffolds at different time points (*i.e.*, at day 1,4 and day 7). In general, all the scaffolds served as biocompatible matrices for the pre-osteoblast cells. This is evident as the constituents of the scaffold have been widely reported to promote cell viability and proliferation. The scaffolds promoted cell viability as seen from day 1, 4, and up to day 7. All the scaffolds showed increased viability at day 7, which shows that these matrices provide a favorable microenvironment for cell viability and proliferation with no significant cytotoxic effects. The viability results clearly illustrated that the presence of silk, HAp, and SA governs a higher percentage of viability, as can be observed in Fig. 13. S2 on day 4 showed increased viability as compared to other scaffolds. On day 7, however, high cell viability was seen in all scaffolds, which is a result of the good cell attachment properties of silk and HAp. Enhanced cell viability was observed in all types of scaffolds on day 7 as compared to day 4 (*p* < 0.0001). High cell viability at day 7 can be associated with degradation of the scaffold. As the matrix degrades, the release of additives such as ZnO, HAp, and SA was increased, hence increasing the cell proliferation and viability (Fig. 13). The results indicate that the scaffolds are non-toxic and biocompatible, and osteoblasts cultured in these scaffolds can attach, spread, and proliferate, which demonstrates their preliminary bone support activity. The biocompatibility of HAp and SF and their role in promoting adhesion and cell proliferation have been reported.^67^

### 3.10 Live-dead assay

**Fig. 14:**
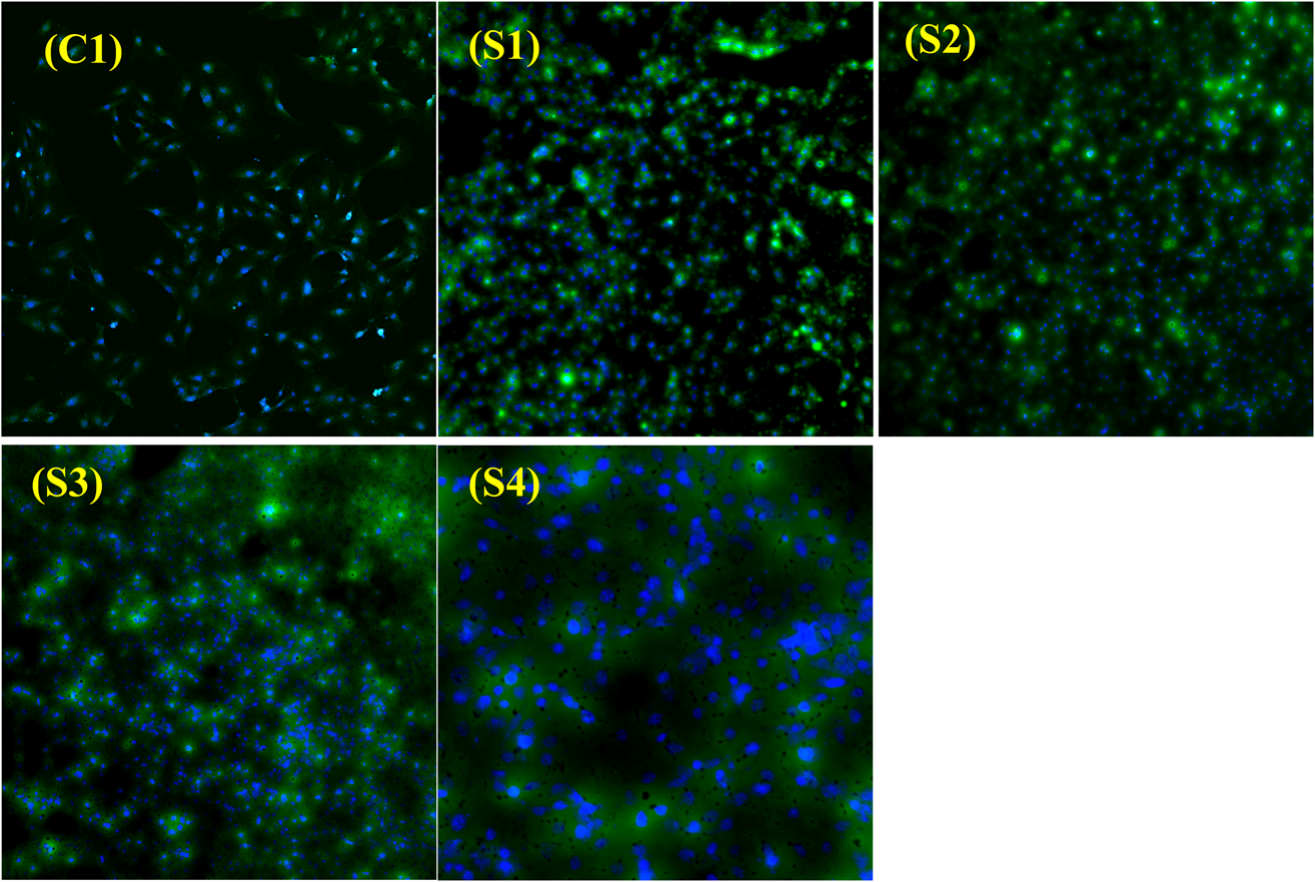
Pre-osteoblast cell viability images (Calcein AM, DAPI, and PI staining) depicting overall viable cells on all of the scaffold surfaces and within the porous structure. The blue colour depicts the nucleus of live cells, whereas the green colour indicates live cells.

Live-dead assay was carried out to evaluate viable and dead cells seeded on the composite scaffolds (see Fig. 14). The cells were seeded on the surface of each scaffold and incubated for 24 hours, followed by staining with calcein AM (stains viable cells), DAPI (stains nuclei of live cells), and propidium iodide (stains dead cells). Representative fluorescent images taken at 10× demonstrated that all scaffolds served as biocompatible matrices for the pre-osteoblast cells. Viable cells (green) and nuclei of live cells (blue) were seen on the scaffold surface, spreading throughout the porous architecture of the matrices. Moreover, no dead cells were seen in the images, which show the biocompatible nature of the synthesized scaffolds. A higher cell density was observed in S2, S3, and S4 scaffolds. The higher number of cells in these scaffolds as compared to C1 is due to the presence of ZnO, HAp and succinic acid in these samples. ZnO is well known for its angiogenic potential. ZnO generates the formation of ROS (reactive oxygen species), which stimulates the formation of new blood vessels, thereby driving cell proliferation.^19,68^ Hydroxyapatite is well-known for its ability to stimulate cell growth owing to its high interfacial adhesion to cells. HAp releases Ca^2+^ ions, which stimulate expression of bone-associated proteins, *i.e.*, sialoprotein (BSP) and osteopontin 69. The role of succinic acid in promoting collagen mineralization has been elucidated before. Modified succinic acid strengthens the capacity of the collagen matrix to attract more calcium ions, ultimately leading to mineralization of collagen fibers.^70^ Silk-based biomaterials, such as the one reported in this research article, can be promising candidates as they support cell attachment and proliferation with no cytotoxicity.^71–73^

## 4 Conclusion

Bone tissue engineering requires biomaterials that closely replicate the native bone microenvironment while also having adjustable physicochemical characteristics and good compatibility with cells. This study investigated the synthesis of composite scaffolds made from silk fibroin (SF), hydroxyapatite (HAp), zinc oxide (ZnO), and succinic acid (SA), which were formed through the freeze-gelation technique, as a potential biomaterial that can support bone growth. The addition of SA as a bio-functional enhancer was crucial in improving the performance of the scaffolds. SA influenced the degradation rate of scaffolds, permitting a controlled and gradual breakdown over time without losing the structural integrity of the scaffolds. Notably, while the concentration of SA affected degradation, the mechanical properties remained mostly unchanged, indicating the potential to fine-tune biological behaviour independently of mechanical strength. Additionally, *in vitro* tests demonstrated the compatibility of synthesized scaffolds with cells, showing high viability of pre-osteoblasts and no toxicity related to the presence of SA. Overall, this research underscores the strategic incorporation of succinic acid to enhance scaffold functionality, presenting a promising and potential biomaterial supporting bone growth. However, further studies, including quantitative cell attachment, osteogenic differentiation assays, mineralization evaluation, gene expression studies, and *in vivo* experiments, will be required to confirm the scaffolds' bone regeneration potential.

## Conflicts of interest

There are no conflicts to declare.

## Data Availability

Data for this article including prism files of original graphs are available at Google Drive. https://drive.google.com/drive/folders/1fWhse2GzUNT8BZ8Uy1ujvN3wPMYNRbYi?usp=drive_link.
